# Doxorubicin-Loaded Metal-Organic Framework Nanoparticles as Acid-Activatable Hydroxyl Radical Nanogenerators for Enhanced Chemo/Chemodynamic Synergistic Therapy

**DOI:** 10.3390/ma15031096

**Published:** 2022-01-30

**Authors:** Honghui Li, Ying Zhang, Lingxia Liang, Jiaxing Song, Zixuan Wei, Shuyue Yang, Yunong Ma, Wei R. Chen, Cuixia Lu, Liewei Wen

**Affiliations:** 1 Medical College, Guangxi University, Nanning 530004, China; sailo1997@163.com (H.L.); zhangy2313@163.com (Y.Z.); xiaotuliang1996@163.com (L.L.); qiangkunxuan@163.com (Z.W.); 18654363051@163.com (S.Y.); mynnong@163.com (Y.M.); 2 Zhuhai Precision Medical Center, Zhuhai People’s Hospital (Zhuhai Hospital Affiliated with Jinan University), Jinan University, Zhuhai 519000, China; 3 Guangdong Key Laboratory for Genome Stability & Disease Prevention, Cancer Research Center, Department of Pharmacology, Shenzhen University Health Science Center, Shenzhen 518000, China; kerphilo@163.com; 4 Stephenson School of Biomedical Engineering, Gallogly College of Engineering, University of Oklahoma, Norman, OK 73019, USA; wei-r-chen@ou.edu

**Keywords:** doxorubicin, chemotherapy, metal–organic framework, hydroxyl radicals, chemodynamic therapy

## Abstract

Doxorubicin (DOX) is a widely used first-line antitumor agent; however, acquired drug resistance and side effects have become the main challenges to effective cancer therapy. Herein, DOX is loaded into iron-rich metal–organic framework/tannic acid (TA) nanocomplex to form a tumor-targeting and acid-activatable drug delivery system (MOF/TA-DOX, MTD). Under the acidic tumor microenvironment, MTD simultaneously releases DOX and ferrous ion (Fe^2+^) accompanied by degradation. Apart from the chemotherapeutic effect, DOX elevates the intracellular H_2_O_2_ levels through cascade reactions, which will be beneficial to the Fenton reaction between the Fe^2+^ and H_2_O_2_, to persistently produce hydroxyl radicals (•OH). Thus, MTD efficiently mediates chemodynamic therapy (CDT) and remarkably enhances the sensitivity of chemotherapy. More encouragingly, the cancer cell killing efficiency of MTD is up to ~86% even at the ultralow equivalent concentration of DOX (2.26 µg/mL), while the viability of normal cells remained >88% at the same concentration of MTD. Taken together, MTD is expected to serve as drug-delivery nanoplatforms and •OH nanogenerators for improving chemo/chemodynamic synergistic therapy and reducing the toxic side effects.

## 1. Introduction

Cancer ranks as a leading cause of death in countries around the world, and the burden of cancer incidence and mortality is rapidly increasing worldwide. Chemotherapy is one important clinical procedure for cancer treatment [1]. However, multidrug resistance and side effects of anticancer drugs have become the major challenges that limit the successful outcome of this option [2,3]. Thus, exploring the method of reducing the strong side effects and the potential molecular mechanisms involved in drug resistance will contribute greatly to increasing the therapy efficiency [4,5]. As an anthracycline antitumor agent, DOX has good clinical efficacy in the treatment of a variety of tumors. DOX can be tightly bound to DNA by embedding between G–C base pairs and destroying its spatial structure in the presence of a considerable concentration of drugs, thereby inhibiting the synthesis of DNA and DNA-dependent RNA, then inhibiting cell transcription and proliferation [6,7]. Despite the extensive and long-term clinical application of DOX, substantial hurdles remain to be addressed, including non-specific delivery, poor solubility, short half-life, evoked multidrug resistance, and other strong side effects. Nowadays, multifunctional nanoparticles have been developed to deliver antitumor drugs through active or passive targeting, such as liposomes and polymer micelles [8]. Nanoparticles-based therapies have gained attention and made gratifying progress in the battle for cancer treatment. As the first FDA-approved anticancer nanodrug, Doxil^®^ (PEGylated liposomal formulation of doxorubicin) was approved for the clinical treatment of ovarian and metastatic breast cancer, as well as various forms of myeloma [9]. Since the success of Doxil in clinical trials, a myriad of studies has been conducted to transform R&D efforts into commercially available products. Unexpectedly, the clinical data did not manifest that Doxil enhanced antitumor efficacy, compared with the upshot of free DOX [10,11]. There still exists an urgent medical demand to improve the efficacy or reduce the toxic side effects of DOX.

Recently, the activatable nanocarriers for drug delivery have attracted extensive attention because of their more specific drug-release property, which remarkably increases the effective concentration of drugs at tumor sites [12]. It is well known that the occurrence and development of malignant tumors result in the special tumor microenvironment (TME), such as hypoxia, low pH, low catalase expression, etc. [13]. Therefore, the studies on activatable nanocarriers that respond to TME and intracellular signals can overcome crucial challenges in conventional nanodrug delivery systems, which will be helpful in enhancing the therapeutic efficacies and reducing the side effects [14]. In particular, the acidic pH of tumor extracellular and intracellular microenvironment is regarded as an appropriate internal trigger for the drug release in the tumor region and/or within the endosomes and lysosomes of tumor cells. Compared with the pH value of normal tissues (pH 7.4), the extracellular pH value of tumor is between 6.0 and 7.2 [15], and the pH value of lysosomes and endosomes of tumor cells decreased to 4.0–5.0 [16]. Metal–organic framework (MOF) nanoparticles with unique physicochemical properties, such as tunable and porous structure, easy functionalization, and large surface areas, had been extensively used for drug delivery, catalysis, and imaging [17,18,19]. MOFs can be biodegradable due to the weak coordination bonds, which definitely permits a promising platform for drug delivery and stimuli-responsive release [19]. MIL-101(Fe)-NH_2_ nanoparticles have been found to have high drug loading efficiency and possess enhanced tumor cell uptake. Moreover, they have been shown to have a pH-responsive release property [20]. In addition, it is worth noting that MIL-101(Fe)-NH_2_ nanoparticles mimicking enzymes are capable of catalyzing H_2_O_2_ in the tumor microenvironment, to generate hydroxyl radical (•OH) through Fenton reaction, and inducing tumor cell apoptosis, which is termed chemodynamic therapy (CDT) [18,21]. Unfortunately, the CDT efficacy is severely limited by H_2_O_2_ supply and iron metabolism in the tumor. Accordingly, it is a greatly necessary and important way to increase the production of •OH by elevating the content of intracellular H_2_O_2_ level and increasing the catalyst iron ions amount in tumors.

**Scheme 1:**
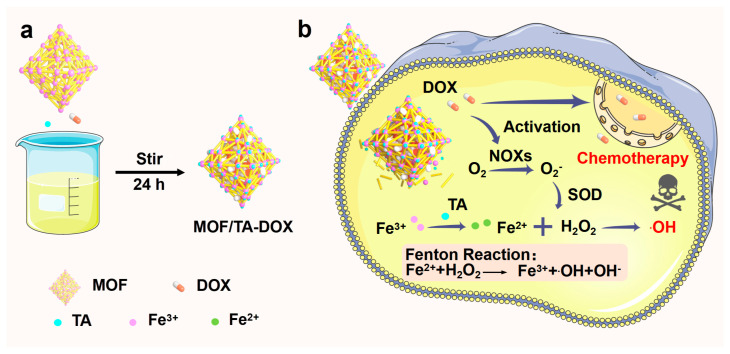
Illustration of MTD as an acid-activatable nanodrugs enhancing tumor chemotherapy: (**a**) preparation of MTD and (**b**) mechanism of MTD enhancing tumor chemo/chemodynamic therapy.

Prior studies have shown that DOX elevates the intracellular H_2_O_2_ level through PARP and NADPH oxidase activation except inducing cell apoptosis through inhibition of topoisomerase II [22,23]. It is believed that DOX can enhance oxidative stress, which is expected to improve the MIL-101(Fe)-NH_2_ nanoparticles-initiated CDT efficacy by promoting the production of •OH. In this study, MIL-101(Fe)-NH_2_ nanoparticles were firstly chelated with a natural iron chelator tannic acid (TA) to form MOF/TA nanoparticles. Then, DOX was loaded into the inner cavities of MOF/TA nanoparticles with high efficiency, benefiting from its porous structure and large surface areas (Scheme 1a). The MOF/TA-DOX nanocomplexes (MTD) as a DOX delivery and acid-activatable release platform could release DOX in the acidic TME, accompanied by the degradation of MOF. Apart from the chemotherapeutic effect, DOX also elevated the content of intracellular H_2_O_2_ levels. Meanwhile, during the degradation process, ferric ion (Fe^3+^) could be rapidly reduced to ferrous ion (Fe^2+^) by TA. Consequently, the intracellular •OH was rapidly and massively produced due to the considerably higher catalytic activity of Fe^2+^ and high content of H_2_O_2_ (Scheme 1b). The drug delivery and catalytic nanoplatform based on the iron-rich metal–organic framework/tannic acid (TA) nanocomplex was expected to furnish an alternative strategy for improving the efficacy of chemotherapy and reducing the side effects.

## 2. Materials and Methods

### 2.1. Chemicals and Materials

Ferric chloride hexahydrate (FeCl_3_•6H_2_O), amino terephthalic acid (BDC-NH_2_), and tannic acid (TA) were acquired from Macklin (Shanghai, China). ICG-NHS was purchased from Ruixibio (Xi’an city, China). Potassium bromide (KBr) was purchased from Meryer (Shanghai, China). Methylene blue (MB) was purchased from Rhawn (Rhawn Co., Ltd., Shanghai, China). ROSGreen H_2_O_2_ probes were purchased from Shanghai Maokang Biotechnology, China. Finally, 2-(4-Amidinophenyl)-6-indolecarbamidine dihydrochloride (DAPI) was purchased from Beyotime Institute of Biotechnology (Shanghai, China).

### 2.2. Characterization

Morphological study of the MOF/TA was performed with transmission electron microscopy (TEM, JEM-1230, JEOL, Akishima, Japan) and scanning electron microscopy (SEM, Hitachi H-7593; Hitachi, Ltd., Tokyo, Japan). FTIR spectroscopy analyses of MOF, TA, and MOF/TA were conducted using an FT-IR spectrophotometer (Nicolet iS5, Thermo Fisher Scientific, Waltham, MA, USA). ESR spectra of different samples with DMPO were recorded on an ESR spectrometer (Bruker EMXnano, Bruker, Bremen, Germany). H&E stains of mice tumors collected from different groups were imaged using an inverted fluorescence microscope (DM IRE2, Leica, Wetzlar, Germany). The hydrodynamic diameters of MOF/TA were determined by dynamic light scattering (DLS, Malvern Zetasizer Nano-ZS ZEN 3600, Malvern Instruments, Worcestershire, UK).

### 2.3. Synthesis and Analysis of MTD Nanoparticles

Typically, 1.8912 g FeCl_3_•H_2_O and 0.6339 g 2-aminoterephthalic acid were placed in a Teflon-lined autoclave, and 43 mL DMF was added. Next, the above solution was stirred at room temperature for 30 min until the chemical compounds were dissolved. Then, the Teflon-lined autoclave was put in an electric drying blower box subjected to solvothermal treatment at 180 °C for 24 h. Through centrifugation (10,500× *g* rpm, 10 min) and washing with DMF three times, the nanoparticles were obtained. Then, the MOF nanoparticles were dried in a vacuum for further use. Afterward, 2 mg MOF nanoparticles were dissolved in 2 mL ddH_2_O, and 0.5 mg TA was added; then, the MOF/TA nanoparticles were obtained by stirring the above mixture at room temperature for 12 h. To obtain MTD, 5 mg DOX was dissolved in 1 mL DMSO and dropwise added in MOF/TA solution (20 mg in 10 mL ddH_2_O) and stirred at 37 °C for 12 h. The products were collected by centrifugation, washed three times with ddH_2_O, and stored in ddH_2_O.

To verify the loading content of DOX, the absorption spectra of MOF/TA, DOX, and MTD solution were measured by a microplate reader at 400–800 nm. Different concentration (0, 5, 25, 50, 80, 100 μg/mL) of DOX solution were prepared to make DOX standard curve.

Loading efficiency (%) = Loaded DOX weight/Added DOX weight × 100(1)

Encapsulation efficiency (%) = Loaded DOX weight/(Nanoparticle weight + Added DOX weight) × 100(2)

### 2.4. Iron Release Analysis

MOF/TA were dispersed in 5 mL acidic (pH = 5.0) and neutral PBS (pH = 7.4), and centrifugated at the indicated time point (0, 1, 2, 4, 8, 12, 24 h). The supernatant (200 μL) was mixed with 200 μL 1,10-phenanthroline monohydrate (0.1 %), and the absorbance at 521 nm was measured using a microplate reader.

To analyze the concentration of iron released from MOF/TA, a standard curve of Fe iron concentration was obtained using different concentrations (0, 5, 25, 50, 80, 100 μg/mL) of FeCl_3_ solution. Next, 200 μL different FeCl_3_ solution was mixed with 200 μL VC and 200 μL 1,10-phenanthroline monohydrate (0.1%), and the absorbance at 521 nm was measured.

### 2.5. Generation of Hydroxyl Radical (•OH)

Methylene-blue colorimetric method was used to analyze the generation of hydroxyl radical. The acidic PBS (pH = 5.0) contained MB (45 μg/mL) was mixed with MOF/TA nanoparticles and H_2_O_2_. After 4 h, the absorbance spectra were measured, and the absorbances at 665 nm were detected. The MB, MB plus H_2_O_2_, and MB plus MOF/TA were set as the control. Generation of hydroxyl radical under neutral conditions was performed in PBS (pH = 7.4) using the same method.

### 2.6. DOX Release from MTD

The release of DOX was analyzed under acidic and neutral conditions. Briefly, 2 mL acidic (pH = 5.0) or neutral (pH = 7.4) PBS containing MTD was put in a dialysis bag (MWCO 3500) and immersed in 10 mL acidic PBS. The DOX concentration was detected with a microplate reader in 2 mL of acidic or neutral solution at a certain time point (0, 1, 2, 4, 8, 12, 24 h), and fresh 2 mL acidic or neutral PBS was used as the control.

### 2.7. Uptake of MTD

To study the internalization of MTD nanoparticles, the nanoparticles were labeled with ICG-NHS. 2 × 10^5^ cells were cultured in 20 mm confocal dishes and grown overnight (12 h). ICG-labeled MTD nanoparticles were co-incubated with the cells for 1 or 4 h and washed with PBS three times. Finally, the cells were stained with DAPI for confocal microscope analysis.

### 2.8. Cellular H_2_O_2_ Content

To study the cellular H_2_O_2_ content of 4T1 cells after MTD treatment, 2 × 10^5^ cells in 20 mm confocal dishes were incubated for 4 h with PBS, MOF/TA, DOX, and MTD nanoparticles, respectively, and the cells were then washed with PBS three times and stained with ROS green H_2_O_2_ probes for confocal microscope analysis.

### 2.9. Fluorescence Imaging for Cellular Fe Ion Content and •OH

Similarly, 2 × 10^5^ cells were incubated for 4 h with PBS, MOF/TA, and MTD nanoparticles, respectively. After being washed with PBS three times, the cells were stained with PGSK probes for confocal microscope analysis to study the cellular Fe ion content of 4T1 cells after MTD treatment. After confocal analysis, the cells were collected for flow cytometry analysis.

Likewise, the cells were incubated with the indicated different treatments and stained with APF probes. The images were obtained by confocal microscope to study cellular •OH generation.

### 2.10. Cell Viability

The CCK-8 assay was applied to measure the toxicity of nanocomplexes in 4T1 cells. The cells (5 × 10^3^ cells per well) were seeded in 96-well plates and grown overnight (12 h) and treated with MOF/TA, DOX, and MTD at 5, 10, 25, 50 μg/mL. After treatment for 24 h, CCK-8 solution (10 μL per well) was added. After incubation for 2 h, the absorbance at 450 nm was detected, and the relative cell viability was calculated.

### 2.11. Animal Experiments

All of the experiments were performed under protocols approved by the Animal Research Ethics Committee of Guangxi University. Balb/c nude female mice (4–5 weeks old) were purchased from Zhuhai BesTest Bio-Tech Co., Ltd. Afterward, 4T1 cells (1 × 10^6^) were inoculated subcutaneously in the right hind thigh of mice. To study the distribution of nanoparticles, 4T1 tumor-bearing mice were injected with ICG and ICG-MTD via the tail vein. When the tumor volume reached about 100 mm^3^, the Balb/c nude mice were imaged at 1, 2, 4, 8, 12, and 24 h after injection by the Living Image IVIS Spectrum (PerkinElmer).

When the tumor volume reached about 100 mm^3^, mice were randomly divided into four groups and treated with PBS, MOF/TA, and MTD (10 mg/kg), respectively. The nanoparticles were injected by the tail vein. During the treatment period, the body weight and tumor sizes (length and width) were measured every three days. The tumor volume was calculated by using the formula: V = L (length) × W (width) × W/2. After treatment, mice were sacrificed, and the tumors and the organs were collected for H&E analysis.

### 2.12. Statistical Analysis

Statistical analyses were conducted using Student’s *t*-tests. Significance was calculated via unpaired two-tailed Student’s *t*-test. *p* value were calculated by *t*-test; * and ** indicated *p* < 0.05 and *p* < 0.01, respectively.

## 3. Results

### 3.1. Preparation and Characterization of Nanodrugs

**Figure 1:**
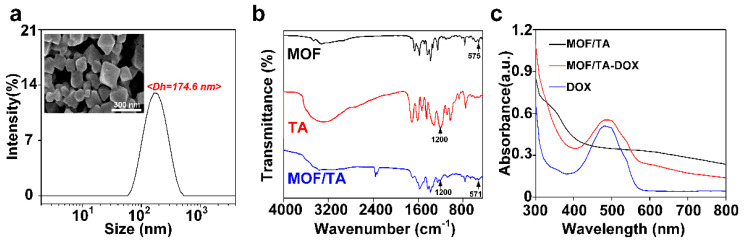
Preparation and synthesis of MTD: (**a**) the determination of particle size and morphology with SEM and DLS; (**b**) FTIR spectra; (**c**) UV-vis spectrum of nanoparticles.

The MOF nanoparticles (MIL-101(Fe)-NH_2_) were prepared according to a previously reported method [24]. The scanning electron microscope (SEM) images of the constructed MOF nanoparticles showed well dispersed and uniform nanoscale size, and the hydrated particle size of MOF was ~174.6 nm (Figure 1a and insert). After stirring with TA, the FT-IR spectrum of MOF/TA showed the characteristic peak at 1200 cm^−1^ and 575 cm^−1^, which were attributed to the polyphenol -OH vibration of TA and Fe-O vibration of MOF, respectively. Meanwhile, the characteristic peak at 1200 cm^−^^1^ also showed weaker than TA due to the coordination of iron and polyphenol -OH groups (Figure 1b). Next, DOX was further stirred with MOF/TA, and the absorbance spectra showed that the characteristic absorption peak of DOX appeared in MTD (Figure 1c). According to the standard curve of DOX, the loading and encapsulation efficiencies were 82.61% and 4.52%, respectively. In addition, the zeta potential of MTD changed from +25.23 mV to −23.36 mV, suggesting the great stability of suspensions. These results indicated the successful preparation of the nanodrugs MTD.

### 3.2. Analysis of Drug Release and •OH Generation

**Figure 2:**
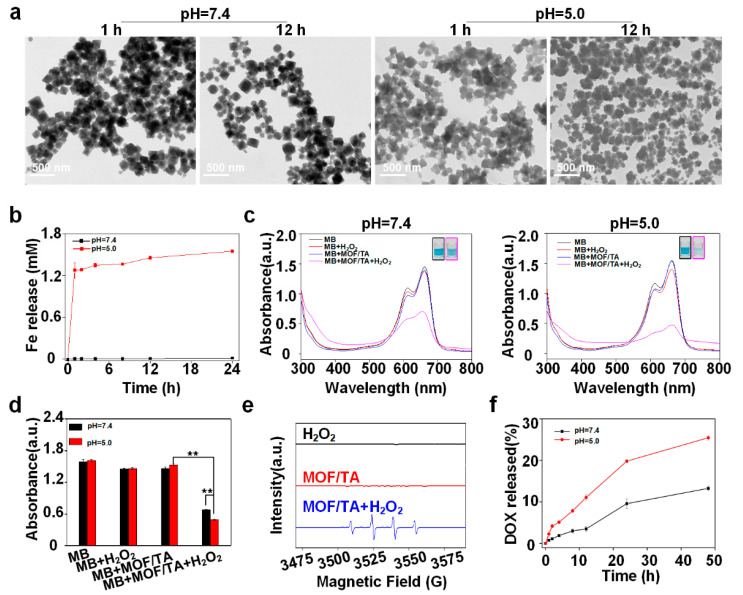
pH-responsive property and •OH generation of MOF/TA: (**a**) pH-responsive biodegraded property of MOF/TA; (**b**) iron release behavior of MOF/TA; (**c**) MB absorbance spectrum under acidic and neutral conditions after the different treatments; (**d**) MB absorbance at 665 nm under acidic and neutral conditions after different treatments; (**e**) ESR spectrum of different samples; (**f**) DOX release behavior of MTD. Data are represented as the arithmetic mean ± SD, and one-way ANOVA followed by Dunnett’s test were performed for statistical analysis (** *p* < 0.01).

Nanoparticles, as drug delivery carriers, could be degraded and release drugs responsively by utilizing the characteristics of the tumor microenvironment, such as weak acidity and high GSH content [25,26]. Therefore, we firstly investigated the biodegradability of MOF/TA nanoparticles. TEM images showed that the MOF/TA nanoparticles displayed a regular square shape at pH = 7.4 and pH = 5.0 for 1 h. However, under the acidic condition, the square shape of MOF/TA nanoparticles changed to round at 12 h, while MOF/TA remained square shape under neutral PBS (Figure 2a), indicating the biodegradability of MOF/TA nanoparticles under acidic conditions. Owing to this characteristic, iron ions could be released from the nanoparticles. Therefore, we next analyzed the release of iron ions by the O-phenanthroline spectrophotometry method. Under acidic conditions, the concentration of iron ions was 1.27624 mM at 1 h, while under neutral conditions, the number of iron ions was 0.00864 mM at 1 h and remained a very low concentration at 24 h, only 0.01811 mM (Figure 2b). These results suggested the rapid release of iron ions from nanoparticles in a pH-dependent manner, which will be beneficial for MOF/TA acting as Fenton reaction catalyzer to generate cytotoxic •OH under acidic tumor microenvironment.

The concept of chemodynamic therapy was firstly proposed by Bu [27], which was dependent on transition metal elements, such as iron, manganese, and copper ions, to catalyze the •OH production through the Fenton reaction. Due to MB oxidization by •OH into colorless solution, an MB colorimetry assay was used to test the ability of MOF/TA nanoparticles to produce •OH. It was observed that the presence of H_2_O_2_ could reduce the absorption value of MB at 665 nm, compared with the MOF/TA group. Notably, compared with the neutral condition, the absorption value of MB under acidic conditions was obviously decreased (Figure 2c,d). This resulted from efficient •OH generation by Fenton reaction since many iron ions were rapidly released from MOF/TA nanoparticles under acidic conditions. Interestingly, MOF/TA alone could reduce the absorption value of MB, which was related to the fact that MB is a kind of electronegative dye and can adsorb to positively charged nanoparticles [28].

Next, electron spin resonance spectroscopy (ESR) was used to verify whether MOF/TA nanoparticles have the ability to catalyze H_2_O_2_, to produce •OH. As shown in Figure 2e, the characteristic peak of •OH could be observed in the presence of MOF/TA and H_2_O_2_, indicating the generation of •OH catalyzed by MOF/TA. The drug release profiles of MTD were further evaluated in different pH conditions. The results showed that the cumulative release of DOX was in a time-dependent way. Compared with the neutral condition, the acidic condition facilitated the release of DOX from the nanoparticles (Figure 2f).

### 3.3. Detection of Intracellular •OH and Cytotoxicity of the Nanodrugs

**Figure 3:**
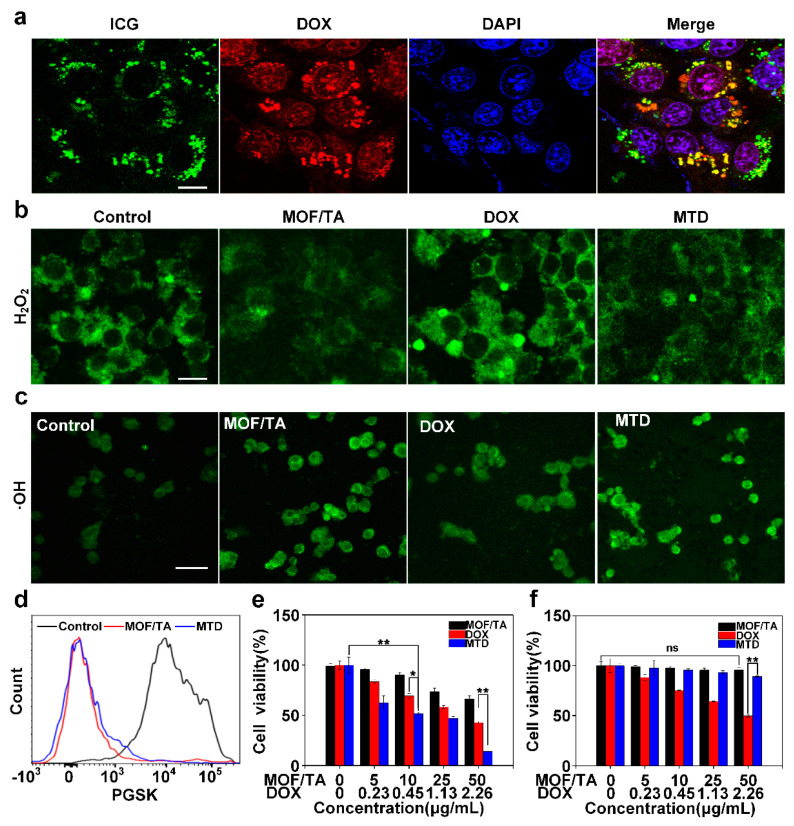
In vitro chemo/chemodynamic therapy effect: (**a**) CLSM images of MTD internalized behavior. Scale bar, 10 μm; (**b**) CLSM images of cellular H_2_O_2_ content after different treatments. Scale bar, 50 μm; (**c**) CLSM images of cellular •OH content after different treatments. Scale bar, 20 μm; (**d**) flow cytometry analysis of cellular iron content after different treatments. The cell viability of (**e**) 4T1 and (**f**) MCF10A after different treatments were assessed with CCK8. Data are represented as the arithmetic mean ± SD, and one-way ANOVA followed by Dunnett’s test were performed for statistical analysis (* *p* < 0.05, ** *p* < 0.01).

To verify if MTD can be internalized by tumor cells and exert chemotherapeutic effect by increasing the H_2_O_2_ levels, the 4T1 cells were co-incubated with ICG-labeled MTD, and the images were obtained by CLSM analysis. As indicated in Figure 3a, strong green fluorescence was observed in the tumor cells after incubation for 4 h, indicating that MTD could enter effectively into the cells. Notably, red fluorescence of DOX could be observed in both the cytoplasm and the nucleus. This result suggested that DOX released from MTD could enter the nucleus, which was consistent with a previous study.

DOX can induce apoptosis as well as increase the content of H_2_O_2_ in cells [29]. Thus, a green fluorescence probe for H_2_O_2_ was used to detect the levels of intracellular H_2_O_2_ content by CLSM after the cells were incubated with the indicated treatments. The results showed that the green fluorescence intensity of DOX was stronger than that of the control group, indicating that the content of H_2_O_2_ in the cells was increased (Figure 3b). Compared with the control, treatment with MOF/TA obviously decreased the green fluorescence intensity. This was due to the consumption of H_2_O_2_ for the Fenton reaction catalyzed by MOF/TA. The presence of DOX could partly invert this inhibitory effect of MOF/TA. These results indicated that DOX could compensate for H_2_O_2_ consumption by MOF/TA to elevate the substrate of Fenton reaction and promote the formation of •OH, improving the sensitivity of chemotherapy.

Considering that MTD could increase the intracellular iron ions that catalyzed the formation of •OH from H_2_O_2_ in tumor cells, and the chemotherapeutic drugs DOX released from MTD could elevate the intracellular H_2_O_2_ content as the substrates for Fenton reaction, we speculated that a considerable amount of cytotoxic •OH could be produced in cells after MTD treatment. To verify this, an APF probe was used to detect the generation of •OH. The images obtained by CLSM showed an increase in fluorescence intensity in cells after treatment with DOX, MOF/TA, and MTD groups (Figure 3c).

Next, the effect of MTD on intracellular iron content was evaluated in tumor cells. A heavy metal fluorescence quenching indicator PGSK was used to indicate the content of iron ions in cells. After treatment with MOF/TA and MTD, the fluorescence intensity of PGSK in cells was significantly quenched, compared with that in the control group (Figure 3d), indicating that there was a high content of ferrous ions in the cells. The high concentration of iron ions in cells, as the catalyst of Fenton reaction, could provide the premise for the conversion of H_2_O_2_ to •OH. Among them, the strongest fluorescence intensity could be observed in cells treated with MTD, indicating that MTD treatment could efficiently induce cellular •OH generation. Therefore, it is believed that MTD nanoparticles act as the •OH nanogenerators to effectively kill tumor cells, resulting from Fenton reaction between intracellular iron originated from MOF- and DOX-induced H_2_O_2_ generation. In order to evaluate the cytotoxic effect of MTD, cell viability was detected after the different treatments by a CCK8 kit according to the manufacturer’s protocol. As indicated in Figure 3e, cell viability was <53% after treatment with 10 μg/mL MTD (the equivalent concentration of DOX is as low as ~0.45 μg/mL), and the viabilities in cells treated with the same concentration of MOF/TA; DOX was still >63%, indicating the synergistic chemotherapeutic effect of MTD. More encouragingly, the cancer cell killing efficiency of MTD was up to ~86%, even at the ultralow equivalent concentration of DOX (2.26 µg/mL), while the viability of normal cells remained >88% at the same concentration of MTD (Figure 3f).

### 3.4. Selective Distribution of Nanodrugs in Tumor

**Figure 4:**
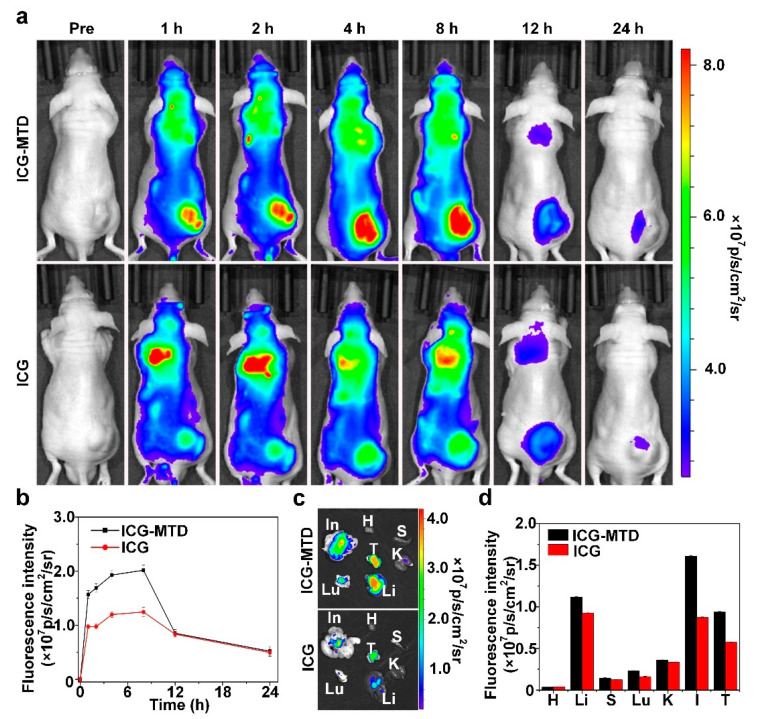
In vivo distribution of MTD: (**a**) fluorescence imaging of 4T1 tumor-bearing mice after tail vein injection with ICG- or ICG-MTD; (**b**) MFI values of the ICG fluorescence following the treatment in (**a**); (**c**) ex vivo fluorescence images of major organs and tumors in mice at 8 h post injection; (**d**) the corresponding MFI analysis at 8 h post injection.

To test if the nanoparticles could be enriched in tumors, ICG- or ICG-labeled MTD were, respectively, injected intravenously to 4T1 tumor-bearing mice. Fluorescence images were obtained by the Living Image IVIS spectrum at the specified time points after injection. Strong fluorescence within the tumor could be detected in both ICG- and ICG-MTD groups at 8 h, indicating selective accumulation of the nanoparticles in the tumor. It should be noted that strong fluorescence could be observed only in the tumor after ICG-MTD treatment, but mice injected with ICG showed strong fluorescence intensity in both the tumor and other organs (Figure 4a,b). These results indicated that ICG-labeled MTD exhibited a good target for tumors, which is beneficial to improve the antitumor effect of chemotherapy. We next analyzed the distribution of MTD in mice by harvesting the major organs and tumors for ex vivo fluorescence imaging at 8 h post injection. Indeed, tumors from the ICG-labeled MTD treated groups showed strong fluorescence, in comparison with the tumors from the free ICG-treated mice (Figure 4c). This result indicates that MTD nanocomplexes have the properties of long-term circulation and tumor-specific accumulation. It could not be ignored that the highest fluorescence signal was observed in the intestine, which suggested the excretion pathway of MTD (Figure 4d).

### 3.5. Antitumor Effect of Nanodrugs In Vivo

**Figure 5:**
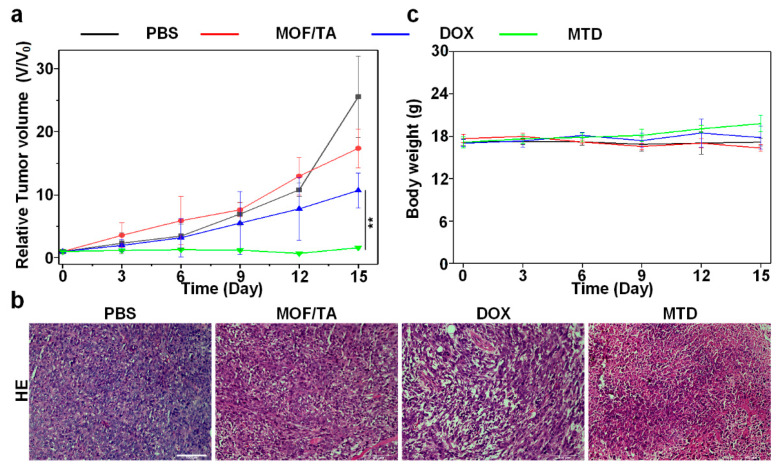
In vivo antitumor effects of MTD: (**a**) relative tumor volume of mice after different treatments; (**b**) H&E analysis of mice tumors collected from different groups. Scale bar, 100 μm; (**c**) body weight of tumor-bearing mice during the treatment period. Data are represented as the arithmetic mean ± SD, and one-way ANOVA followed by Dunnett’s test were performed for statistical analysis (** *p* < 0.01).

To evaluate the antitumor effects of nanoparticles in mice, mice bearing 4T1 tumors were divided into four different treatment groups: PBS, MOF/TA, DOX, and MTD. After injection, tumor growth and body weight of mice were monitored every three days. As shown in Figure 5a, the average tumor size in MOF/TA, DOX, and MTD groups was smaller than that of the control group, indicating their inhibition effects on tumor growth. Treatment with MTD showed the highest inhibitory rate of 93.728% on tumor growth, which was attributed to the synergistic chemotherapeutic effects of nanoparticles. Meanwhile, the body weights of mice in each treatment group did not show an obvious change (Figure 5b), indicating that the nanoparticles have good biological safety. In order to further study the killing effect of nanoparticles on the tumor, tumor tissues of each treatment group were collected after the treatment period, and H&E was performed (Figure 5c). Obvious damage could be observed in tumor tissue from the mice treated with MTD. These results demonstrate that the MTD nanoparticles have good tumoricidal effects and biological safety through selectively targeting tumors and effectively inducing tumor damage.

## 4. Discussion

Currently, two drug delivery nanosystems including liposomes and albumin nanoplatforms had already been approved by the FDA for clinical application [30]. However, the drug delivery to the tumor site relied considerably on the enhanced permeability and retention (EPR) effect, which was due to the defective blood vasculature of tumor tissues, poor lymphatic drainage, and increased vessel permeability [31]. It was only considered as a passive targeting strategy for drug delivery. In this study, although MTD could enhance the effective concentration of drugs at the tumor site by responsive release, it showed poor passive targeting and was easily captured by the reticuloendothelial system, while the chemical bonds on the surface of nanoparticles can be used to link with small molecules such as HA and anti-CD47 to bind receptors that are highly expressed on the surface of tumor cells to achieve active targeting of tumors. [32,33] Therefore, the targeting properties of nanodrugs would be further improved by surface modification of specific targeted molecules. In addition, the •OH production performance was also affected by the limited residence time of nanodrugs in the tumor site. Although the •OH was highly cytotoxic, the short duration is an obstacle to further improving the therapeutic efficacy of tumors. Surprisingly, the strategies of delicately designed nanoparticles may help to solve these difficulties. A previous study shows that, due to their small size, nanoparticles self-aggregate in a tumor microenvironment with high levels of GSH, thus enhancing tumor retention and accumulation of the nanoparticles [34]. It has also widely been reported that the mechanism by which nanoparticles degrade within the tumor microenvironment accompanies the release of the loaded catalytic enzymes, such as poly(ethylene glycol)-modified glucose oxidase (GOx), to continuously generate •OH and enhance the killing effect of tumor cells [35,36]. In addition, the •OH produced by the Fenton reaction between H_2_O_2_ and Fe^3+^/Fe^2+^ can initiate ferroptosis to enhance the killing effect [37]. Recently, immunotherapy, as an emerging strategy in tumor therapy, was expected to specifically inhibit tumors in the long term [38]. To date, numerous immunotherapeutic agents have been approved by FDA including checkpoint inhibitor drugs for targeting the PD-1/PD-L1 axis and cytotoxic T-lymphocyte-associated antigen 4 (CTLA-4) [39]. Accumulating lines of evidence have also suggested chemotherapy combined with immune checkpoint inhibitors would enhance antitumor effects in tumor therapy [40,41]. •OH could trigger immunogenic cell death (ICD) and initiate a potentiated antitumor immune response, as reported in [42,43]. In previous research, we have verified that the •OH nanogenerator enhances the antitumor immune response by inducing ICD in combination therapy with the PD-L1 blockade, efficiently inhibiting tumor growth [21]. Therefore, the combination of immunotherapy represented by immune checkpoint blockers (ICBs) with the nanodrugs mediated chemotherapy could be expected to achieve synergistic enhancement and even long-term antitumor effects.

## 5. Conclusions

In summary, we successfully prepared a pH-responsive, iron-based nanoparticle (MTD), which contributes to enhancing the chemotherapeutic effect and reducing the side effects significantly. MTD increased the effective concentration of DOX at the tumor site and elevated the intracellular H_2_O_2_ concentration based on the characteristics of acidic responsive release and the dual functions of DOX. Accordingly, MTD demonstrated the best therapeutic effect in the 4T1 tumor model, with the highest inhibitory rate of 93.728%, due to the chemotherapeutic effect and property of persistently producing •OH. Taken together, the drug-loaded, iron-rich MOF nanoparticles are promising to serve as activatable nanodrugs for reducing the toxic side effects of chemotherapy and improving the efficacy of tumor chemotherapy.
